# Human metapneumovirus activates NOD-like receptor protein 3 inflammasome via its small hydrophobic protein which plays a detrimental role during infection in mice

**DOI:** 10.1371/journal.ppat.1007689

**Published:** 2019-04-09

**Authors:** Vuong B. Lê, Julia Dubois, Christian Couture, Marie-Hélène Cavanagh, Olus Uyar, Andres Pizzorno, Manuel Rosa-Calatrava, Marie-Ève Hamelin, Guy Boivin

**Affiliations:** 1 Infectious Disease Research Centre, Department of microbiology-Immunology, Laval University, Quebec City, Quebec, Canada; 2 Laboratoire de Virologie et Pathologie Humaine—VirPath team, Centre International de Recherche en Infectiologie (CIRI), INSERM U1111, CNRS UMR5308, ENS Lyon, Université Claude Bernard Lyon, Université de Lyon, Lyon, France; 3 Quebec Heart and Lung Institute, Department of Anatomopathology and cytology, Laval University, Quebec City, Quebec, Canada; Thomas Jefferson University, UNITED STATES

## Abstract

NOD-like receptor protein 3 (NLRP3) inflammasome activation triggers caspase-1 activation-induced maturation of interleukin (IL)-1β and IL-18 and therefore is important for the development of the host defense against various RNA viral diseases. However, the implication of this protein complex in human metapneumovirus (HMPV) disease has not been fully studied. Herein, we report that NLRP3 inflammasome plays a detrimental role during HMPV infection because NLRP3 inflammasome inhibition protected mice from mortality and reduced weight loss and inflammation without impacting viral replication. We also demonstrate that NLRP3 inflammasome exerts its deleterious effect via IL-1β production since we observed reduced mortality, weight loss and inflammation in IL-1β-deficient (IL-1β^-/-^) mice, as compared to wild-type animals during HMPV infection. Moreover, the effect on these evaluated parameters was not different in IL-1β^-/-^ and wild-type mice treated with an NLRP3 inflammasome inhibitor. The production of IL-1β was also abrogated in bone marrow derived macrophages deficient for NLRP3. Finally, we show that small hydrophobic protein-deleted recombinant HMPV (HMPV ΔSH) failed to activate caspase-1, which is responsible for IL-1β cleavage and maturation. Furthermore, HMPV ΔSH-infected mice had less weight loss, showed no mortality and reduced inflammation, as compared to wild-type HMPV-infected mice. Thus, NLRP3 inflammasome activation seems to be triggered by HMPV SH protein in HMPV disease. In summary, once activated by the HMPV SH protein, NLRP3 inflammasome promotes the maturation of IL-1β, which exacerbates HMPV-induced inflammation. Therefore, the blockade of IL-1β production by using NLRP3 inflammasome inhibitors might be a novel potential strategy for the therapy and prevention of HMPV infection.

## Author summary

Human metapneumovirus (HMPV), a negative-stranded, enveloped RNA virus, is recognized as one of the leading causes of acute respiratory disease in children since its discovery in 2001. Nevertheless, there is currently no licensed vaccine for the prevention of HMPV infection and treatment modalities are limited to the use of ribavirin, a weak antiviral agent or immunoglobulins. NOD-like receptor protein 3 (NLRP3) inflammasome has been shown to be involved in the pathogenesis of several RNA viral diseases but its role during HMPV infection has not been fully studied. Here, we report for the first time that NLRP3 inflammasome is activated by the small hydrophobic protein of HMPV, leading to the release of IL-1β, which has the potential to exacerbate inflammation. However, NLRP3 inflammasome has no direct influence on viral replication. Thus, IL-1β-mediated inflammatory process plays an important role during HMPV infection and, therefore, anti-IL-1β strategies such as the use of NLRP3 inhibitors may be a novel potential approach for the prevention and therapy of HMPV disease.

## Introduction

The inflammasomes are cytosolic multiprotein complexes responsible for caspase-1 activation [1]. Once activated, caspase-1 proteolytically cleaves interleukin (IL)-1β and IL-18 precursors (pro-IL-1β and pro-IL-18), leading to the release of mature forms [2, 3]. Among identified inflammasomes, the NOD-like receptor protein 3 (NLRP3) inflammasome containing NLRP3, adapter protein apoptosis-associated speck-like protein (ASC) and pro-caspase-1 is the most fully studied [4]. NLRP3 inflammasome activation is a two-step process. The first step involves a priming signal provided by microbial molecules or endogenous cytokines, which upregulates the transcription of inactive NLRP3, pro-IL-1β and pro-IL-18. The second step is characterized by the oligomerization of NLRP3 and subsequent assembly of NLRP3, ASC and pro-caspase-1 into a complex [5]. This signal is provided by numerous stimuli such as ATP, pore-forming toxins, viral RNA, etc. Most of them induce potassium efflux, calcium signaling, reactive oxygen species generation, mitochondrial dysfunction and lysosomal rupture [6].

The NLRP3 inflammasome has been demonstrated to be activated by many RNA viruses and could play distinct roles during viral infections [7, 8]. NLRP3 inflammasome activation has been reported to aggravate Newcastle virus, murine hepatitis virus, coxsackievirus B3, Dengue virus, and Zika virus infections [9–13] but exerts beneficial effects to the host response against enterovirus 71 and rabies virus diseases [14, 15]. Surprisingly, although NLRP3 inflammasome could be activated by vesicular stomatitis and encephalomyocarditis viruses, it seemed to have no influence on the pathogenesis of these two viruses [16]. In the case of influenza A virus infection, several studies investigating the role of NLRP3 inflammasome have yielded controversial results [17–20], leading to the conclusion that NLRP3 inflammasome might play a dual protective or detrimental role at different stages of influenza A virus infection [21].

Human metapneumovirus (HMPV) is a member of the *Metapneumovirus* genus within the new *Pneumoviridae* family of non-segmented, negative-stranded, enveloped RNA viruses [22]. This virus is one of the leading causes of respiratory tract disease in both children and adults. The adaptive immune response generated against HMPV is usually inefficient at protecting from reinfections, which are repeated throughout life [23]. There is currently no licensed vaccine to prevent HMPV infection and its treatment is still limited to the use of ribavirin, a weakly effective antiviral agent, and immunoglobulins [24]. Thus, highlighting the role of NLRP3 inflammasome during HMPV infection may provide a new perspective on the prevention and treatment of this viral disease. To date, only one study has reported increases in the production of IL-1β and IL-18, accompanied by an upregulation of NLRP3 mRNA expression in HMPV-infected children, as compared to control healthy individuals [25]. However, the authors did not clarify the role of NLRP3 inflammasome during HMPV infection.

In the current study, by using a pharmacological approach, small hydrophobic protein-deleted recombinant HMPV (HMPV ΔSH) as well as IL-1β-deficient (IL-1β^-/-^) mice, we show that NLRP3 inflammasome can be activated by HMPV SH protein. Once activated, this multiprotein complex exerts a deleterious effect during HMPV infection in mice by triggering IL-1β release. Therefore, targeting NLRP3 inflammasome as well as IL-1β may be of interest for the development of new therapeutics against HMPV infections.

## Results

### MCC950-induced NLRP3 inflammasome inhibition prevents IL-1β and IL-18 production but does not impact HMPV replication *in vitro*

**Fig 1:**
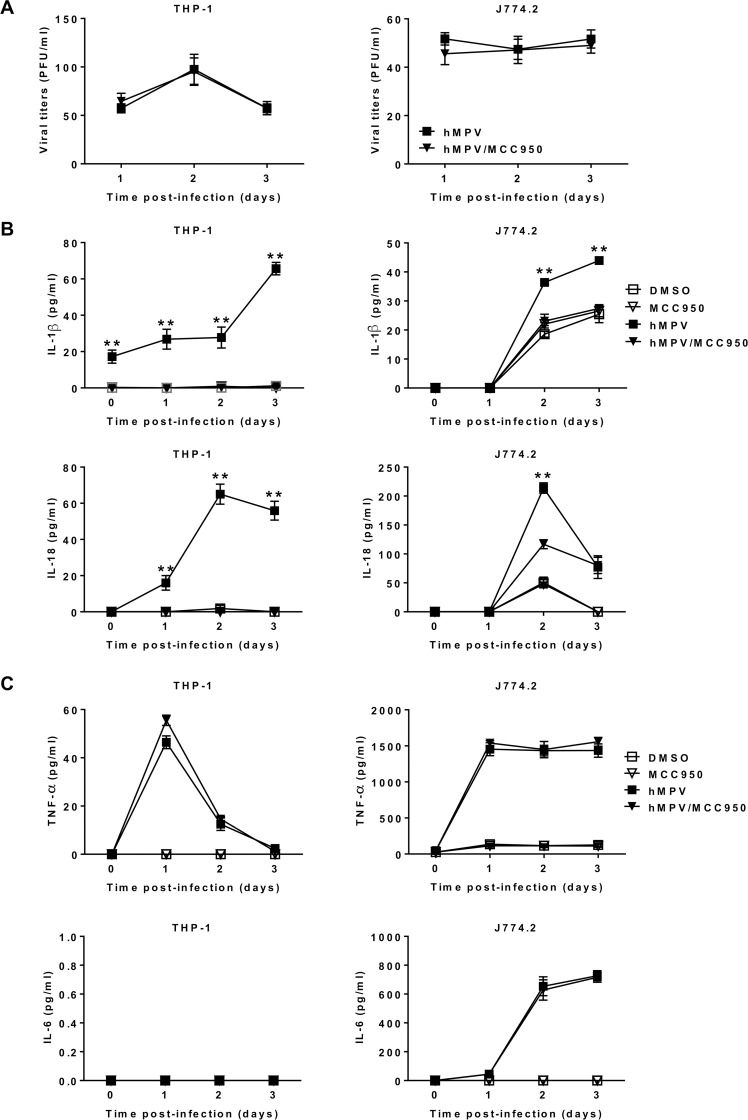
NLRP3 inflammasome activation triggers IL-1β and IL-18 production in HMPV-infected THP-1 and J774.2 cells but has no effect on the viral replication. THP-1 cells were differenciated into macrophages using PMA (100 ng/ml). THP-1 or J774.2 cells were treated with 10 μM of MCC950 or DMSO (control) and then infected or not with HMPV at a MOI of 0.001 (THP-1) or 0.01 (J774.2). (A) The viral titers were determined by immunostaining. Data were collected from three independent experiments. Values were shown as mean ± S.E.M (unpaired Student t-test, n = 5 per group per experiment). (B & C) Cytokine levels were determined in the cell supernatants by ELISA assay. Values are shown as mean ± S.E.M (Kruskal-Wallis test, ** *P* < 0.01 HMPV/MCC950 *vs* HMPV, n = 5 per group).

MCC950 has been recently synthesized and recognized as a specific inhibitor of NLRP3, but not NLRP1, NLRC4 or AIM2 inflammasomes [26]. Since then, it has been preferentially used in various models of NLRP3-related diseases [27]. A recent study has reported that MCC950 did not impact the viability and proliferation of high-glucose-induced human retinal endothelial cells at a concentration of 100 μM [28]. In the current study, we also showed that this inhibitor was safe and usable for both human THP-1 (CC_50_ > 250 μM) and murine J774.2 (CC_50_ > 125 μM) cells (S1 Fig). To determine if NLRP3 inflammasome impacts HMPV replication, THP-1 or J774.2 cells were treated or not with 10 μM of MCC950 because this dose has been shown to be able to block NLRP3 activation in mouse bone marrow derived macrophages, human monocyte derived macrophages and human peripheral blood mononuclear cells [26], and then infected with HMPV. The viral loads evaluated on days 1, 2 and 3 post-infection did not differ between MCC950-treated and control DMSO-treated groups (Fig 1A). We also observed that viral titers were relatively low, but as expected since no trypsin was added during the cell culture assay. Thus, NLRP3 inflammasome had no influence on HMPV replication *in vitro*.

To investigate if NLRP3 inflammasome is responsible for IL-1β and IL-18 production, THP-1 or J774.2 cells were treated or not with MCC950 and then infected or not with HMPV. We found that NLRP3 inflammasome inhibition suppressed IL-1β and IL-18 secretion in THP-1 cells and significantly decreased their concentrations in J774.2 cells (Fig 1B). We also confirm that NLRP3 is responsible for the maturation of only IL-1β and IL-18 [29], as evidenced by no difference in IL-6 and TNF-α levels between MCC950-treated and control groups during HMPV infection (Fig 1C). Of note, we observed no IL-6 production in THP-1 cells upon HMPV inoculation. In order to confirm those results, we used wild-type (WT) bone marrow derived macrophages (BMDM) and NLRP3 KO BMDM cell lines and observed that HMPV-infected BMDM cells induced the production of IL-1β at 48 h, but not HMPVΔSH-infected BMDM (S2 Fig). Conversely, no IL-1β was detected in NLRP3 KO BMDM cells following HMPV infection. Notably, TNF-α was detected in both WT and NLRP3 KO BMDM cells.

### MCC950-induced NLRP3 inflammasome inhibition protects mice against HMPV disease

**Fig 2:**
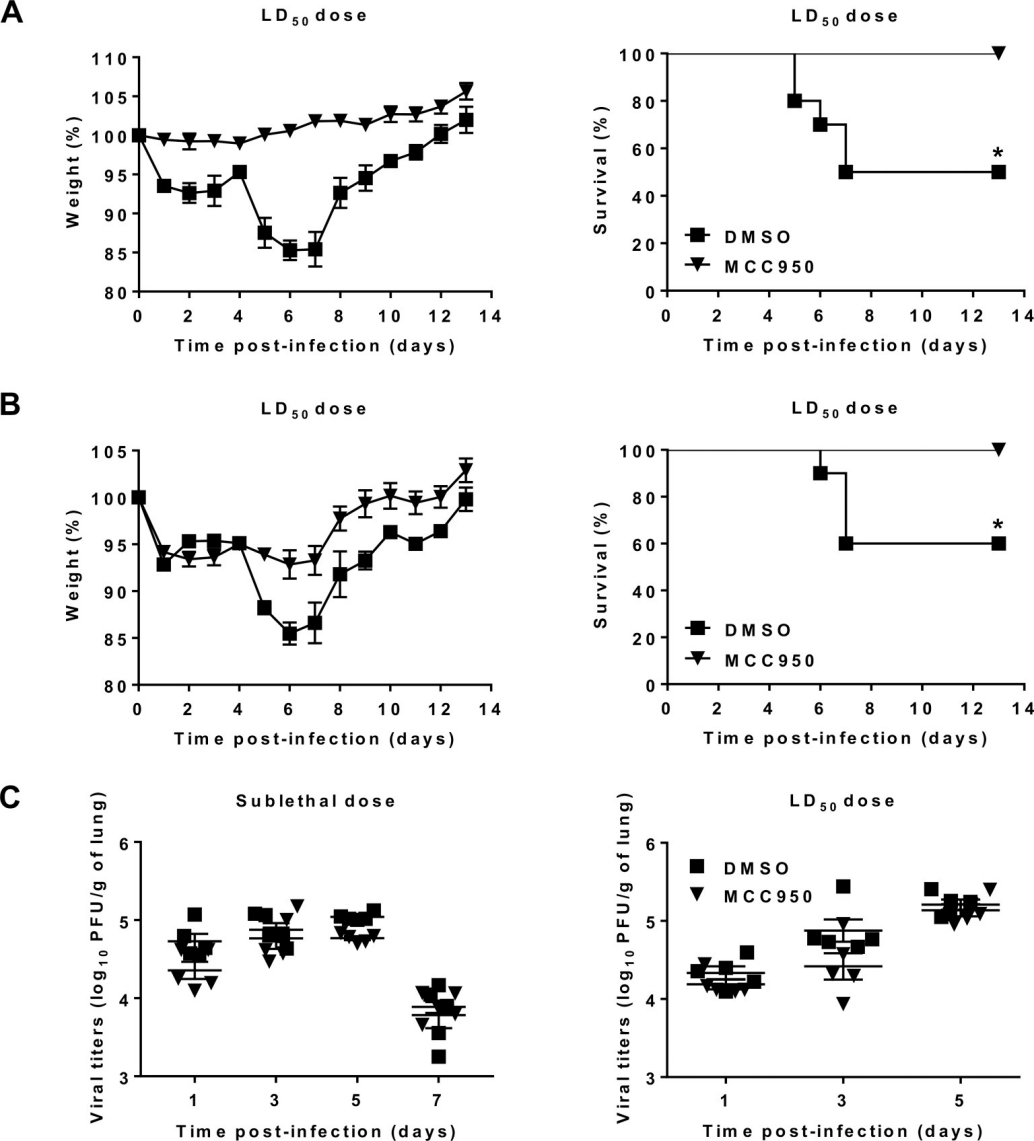
NLRP3 inflammasome contributes to the pathogenesis of HMPV. BALB/C mice were inoculated with HMPV at a LD_50_ dose (5 x 10^5^ PFU per mouse). MCC950 treatment (5 mg/kg) was carried out (A) at the same time or (B) 24 h post-infection and repeated for the next two days (1 time/day). Mice were monitored for survival and weight loss for 14 days after infection and euthanized if humane end points were reached (≤ 20% of initial weight). Values were shown as mean of weight ± S.E.M or average percent survival (Kaplan Meier survial curves, * *P* < 0.05 MCC950 *vs* DMSO, n = 10 per group). (C) Mice were inoculated with HMPV at sublethal (3 x 10^5^) or LD_50_ doses (5 x 10^5^ PFU per mouse) in the presence or absence of MCC950 (5 mg/kg). MCC950 treatment was repeated for the next two days (1 time/day). The viral titers were evaluated in the lung homogenates. Values are shown as mean ± S.E.M (Mann-Whitney U-test, n = 5 per group).

To investigate if NLRP3 inflammasome is involved in the pathogenesis of HMPV, BALB/c mice were treated with MCC950 and infected with HMPV at a LD_50_ dose. No mortality and a less important weight loss were observed in MCC950-treated groups, as compared to controls (Fig 2A). Nevertheless, the protective effect of MCC950 treatment was slightly decreased when treatment was delayed 24 h post-infection (Fig 2B), in comparison with immediate treatment (Fig 2A). Thus, NLRP3 inflammasome plays a detrimental role during HMPV infection and the blockade of its activation may be useful not only for the prevention but also for the therapy against HMPV disease, at least in mice.

We then investigated the effect of NLRP3 inflammasome on HMPV replication by determining viral loads in the lungs. In agreement with *in vitro* results, mice inoculated with HMPV at sublethal or LD_50_ doses both showed no difference in lung viral titers between MCC950-treated and control groups (Fig 2C). Therefore, it is likely that the involvement of NLRP3 inflammasome in the pathogenesis of HMPV does not occur via a direct viral replication-related pathway.

### MCC950-induced NLRP3 inflammasome inhibition attenuates inflammation in HMPV-infected mice

**Fig 3:**
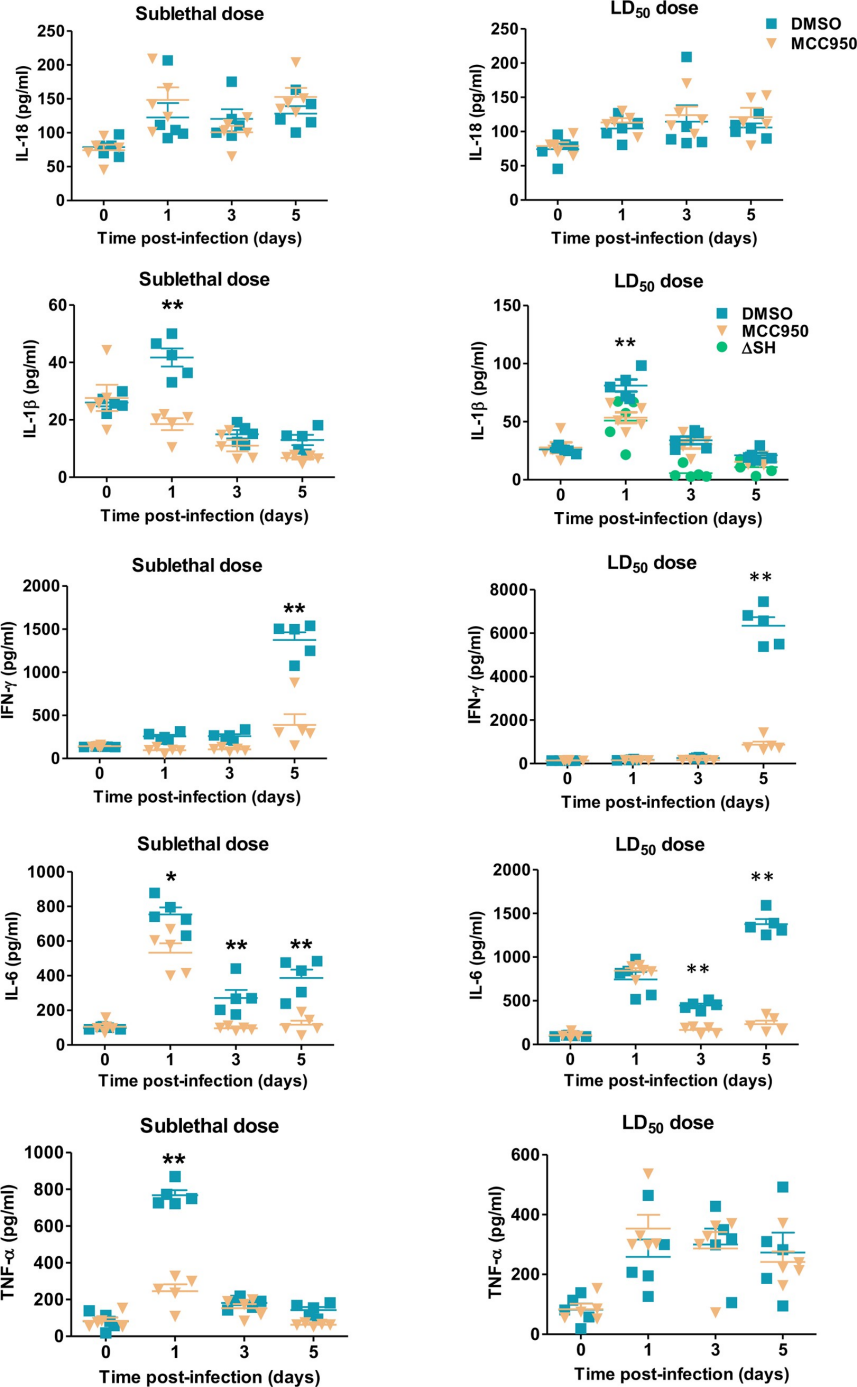
NLRP3 inflammasome contributes to the production of inflammatory cytokines in HMPV-infected mice. BALB/c mice were inoculated with HMPV at a sublethal (3 x 10^5^) or LD_50_ doses (5 x 10^5^ PFU per mouse) in the presence or absence of MCC950 (5 mg/kg). MCC950 treatment was repeated for the next two days (1 time/day). Cytokine levels were measured in the BAL. Values are shown as mean ± S.E.M (Mann-Whitney U-test, * *P* < 0.05; ** *P* < 0.01 MCC950 *vs* DMSO, n = 5 per group). ΔSH group is compared to DMSO.

**Fig 4:**
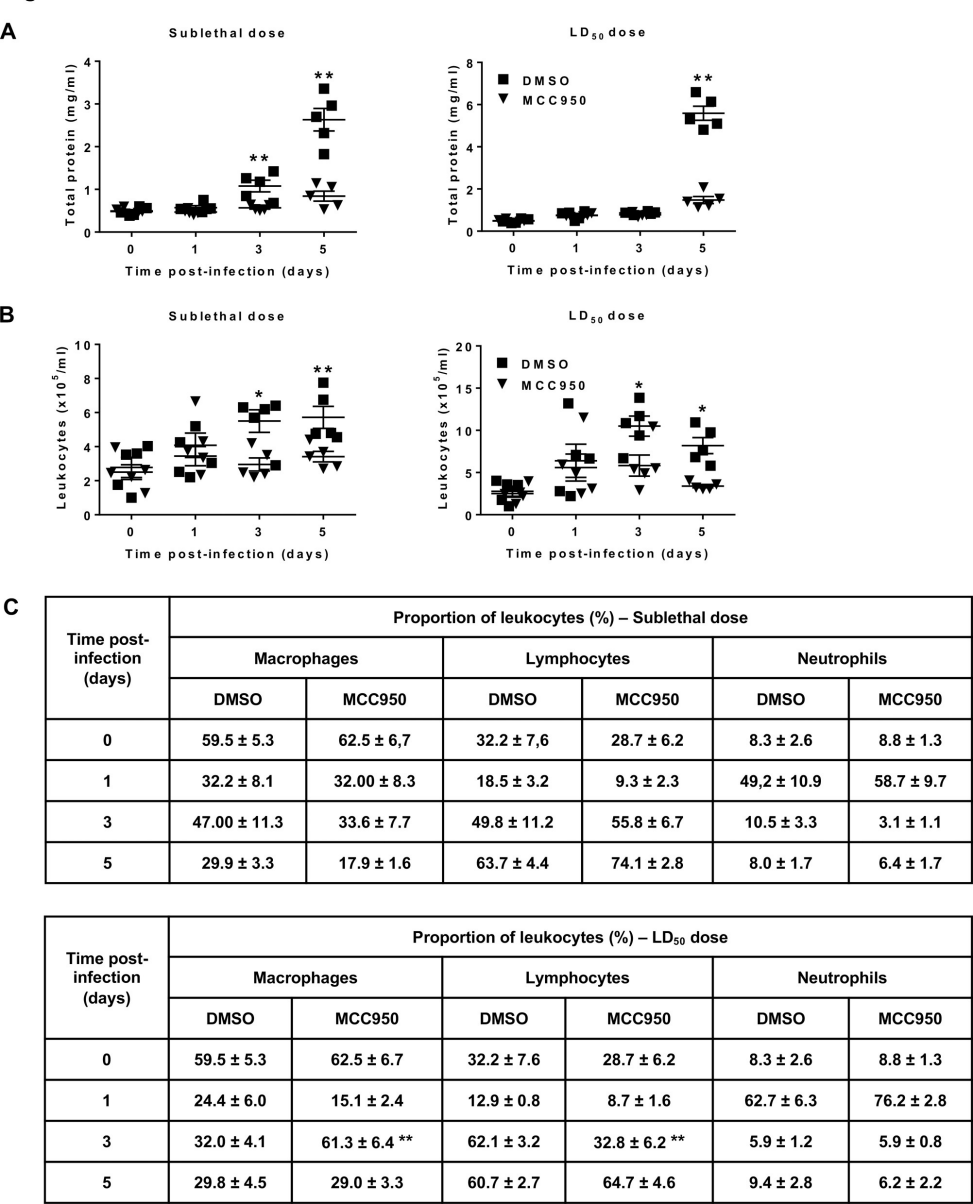
NLRP3 inflammasome contributes to inflammation in HMPV-infected mice. BALB/c mice were inoculated with HMPV at at sublethal (3 x 10^5^) or LD_50_ doses (5 x 10^5^ PFU per mouse) in the presence or absence of MCC950 (5 mg/kg). MCC950 treatment was repeated for the next two days (1 time/day). (A) total protein, (B) leukocyte recruitment and (C) cell differentiation were evaluated in BAL. Values are shown as mean ± S.E.M (Mann-Whitney U-test, * *P* < 0.05; ** *P* < 0.01 MCC950 *vs* DMSO, n = 5 per group).

Because NLRP3 inflammasome did not impact HMPV replication, we hypothesized that it possibly exerts a deleterious effect via IL-1β and/or IL-18-dependent pathways. To verify this hypothesis, we measured IL-1β, IL-18 and other cytokine levels in BAL at different time points from BALB/c mice infected with HMPV at sublethal or LD_50_ doses and treated or not with MCC950. IL-18 levels were not different between MCC950-treated and DMSO-treated mice. By contrast, IL-1β levels in HMPV-infected mice were significantly decreased upon MCC950 treatment on day 1 post-infection (Fig 3). As presented above, NLRP3 inflammasome did not impact HMPV-induced IL-6 and TNF-α secretion *in vitro*. By contrast, interferon (IFN)-γ, IL-6, and TNF-α levels in BAL were significantly decreased upon MCC950 treatment (Fig 3). In parallel, NLRP3 inflammasome inhibition also reduced the alteration of pulmonary capillary permeability and leukocyte recruitment, as evidenced by significant decreases in total protein levels and cell number in BAL from MCC950-treated mice compared to controls during HMPV infection (Fig 4A and 4B).

Thus, NLRP3 inflammasome inhibition protects mice against HMPV disease by exerting an anti-inflammatory effect. Moreover, this anti-inflammatory effect seems to be virus dose-dependent because NLRP3 inflammasome inhibition decreased more efficiently inflammatory parameters in the case of sublethal dose than LD_50_ dose of virus. Indeed, MCC950 treatment induced significant decreases in IL-6 and TNF-α levels on day 1 post-infection and total protein levels on days 3 post-infection (sublethal dose) and 5 post-infection (sublethal and LD_50_ doses) (Figs 3 and 4A).

We then investigated if NLRP3 inflammasome impacts the recruitment of particular cell type(s). Only lymphocyte percentage was decreased on day 3 post-infection upon MCC950 treatment accompanied by an increase in macrophage percentage in mice inoculated with HMPV at a LD_50_ dose (Fig 4C). Lymphocytes decrease on day 3 was characterized by a reduction in % of B and CD8 T cells (S3 Fig). A time-dependent different contribution of each cell type during HMPV infection was detected, as evidenced by the predominance of polymorphonuclear neutrophils on day 1, and then lymphocytes on days 3 and 5 post-infection.

### NLRP3 inflammasome plays a detrimental role during HMPV infection through IL-1β-mediated inflammatory process

**Fig 5:**
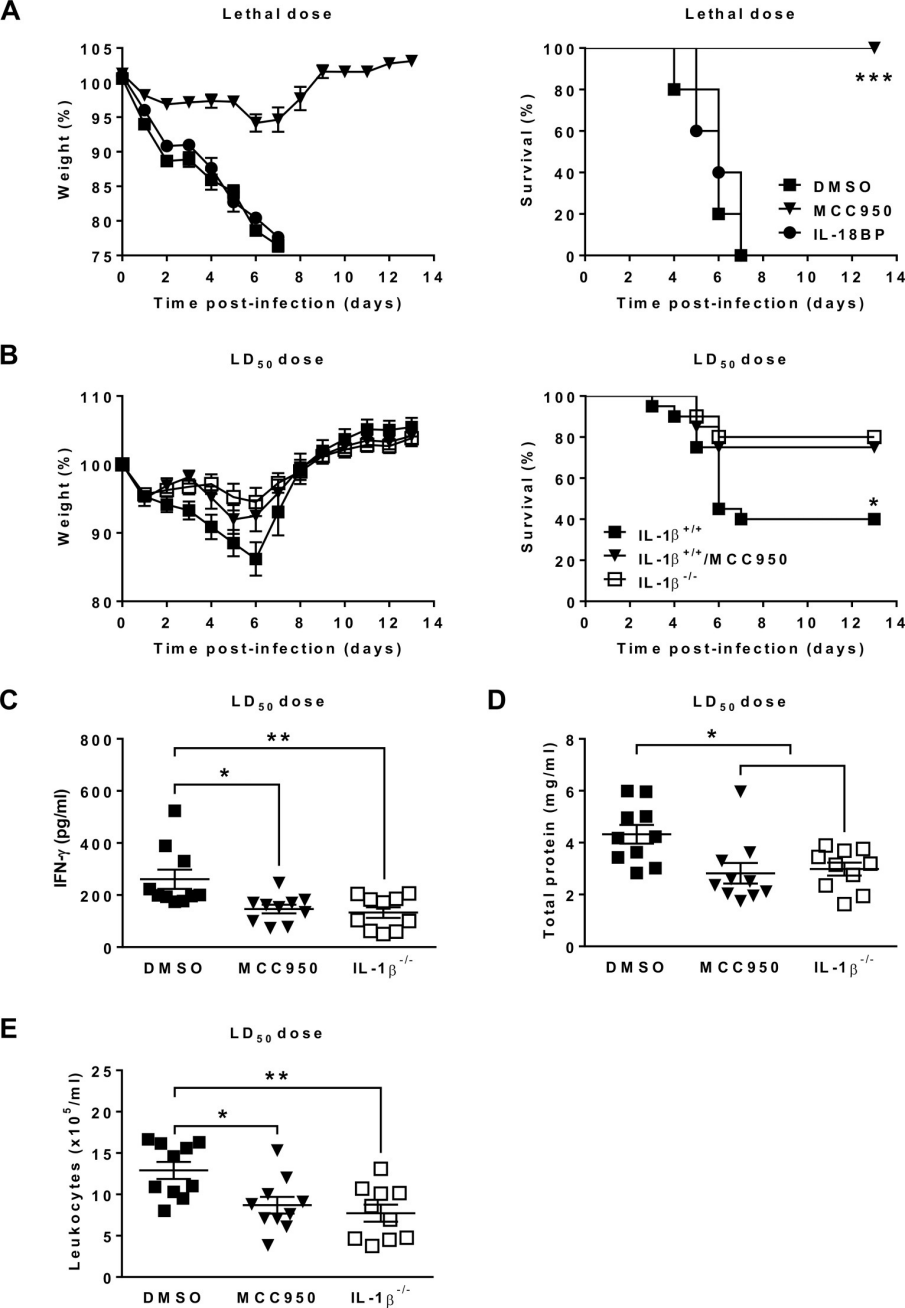
NLRP3 inflammasome activation plays a crucial role in the pathogenesis of HMPV via IL-1β release. (A) BALB/c mice were inoculated with HMPV at a lethal dose (10^6^ PFU per mouse) in the presence or absence of MCC950 (5 mg/kg). Immediately after infection, they were injected or not with IL-18BP (75 μg/kg). MCC950 and IL-18BP treatments were repeated for the next two days (1 time/day). The animals were monitored for survival and weight loss for 14 days after infection and euthanized if humane end points were reached (≤ 20% of initial weight). Values are shown as mean of weight ± S.E.M or average percent survival (Kaplan Meier survial curves, *** *P* < 0.001 MCC950 *vs* DMSO, n = 10 per group). (B) IL-1β^-/-^ and C57BL/6 (IL-1β^+/+^) mice were inoculated with HMPV at a LD_50_ dose (2 x 10^6^ PFU per mouse) in the presence or absence of MCC950 (5 mg/kg). MCC950 treatment was repeated for the next two days (1 time/day). The animals were monitored for survival and weight loss for 14 days after infection and euthanized if humane end points were reached (≤ 20% of initial weight). Values are shown as mean of weight ± S.E.M or average percent survival (Kaplan Meier survial curves, * *P* < 0.05 IL-1β^-/-^ or IL-1β^+/+^/MCC950 *vs* IL-1β^+/+^, n = 20 per group). (C-E) IFN-γ, total protein and leukocyte recruitment were evaluated in BAL of IL-1β^-/-^ and C57BL/6 (IL-1β^+/+^) mice. Values are shown as mean ± S.E.M (ANOVA followed by Tukey post hoc, * *P* < 0.05; ** *P* < 0.01 IL-1β^-/-^ or IL-1β^+/+^/MCC950 *vs* IL-1β^+/+^, n = 10 per group).

Since IL-18 levels were unaltered during *in vivo* infection, we further determined if this cytokine exerts some effects during HMPV disease. IL-18 was therefore inhibited in HMPV-infected BALB/c mice by using IL-18 binding protein (IL-18BP), which functions as an IL-18 antagonist by binding to IL-18 and blocking its biological activities [30–32]. No difference in survival and weight loss between IL-18BP-treated and non-treated mice was observed during HMPV infection (Fig 5A). Thus, the involvement of NLRP3 inflammasome in the pathogenesis of HMPV is IL-18-independent. Interestingly, we noticed that NLRP3 inflammasome inhibition always protected mice against HMPV disease even if virus was administered at a lethal dose, as evidenced by no mortality and slight weight loss (<10% initial weight) in MCC950-treated compared to DMSO-treated mice. This finding enables us to suggest that NLRP3 inflammasome is essential for the pathogenesis of HMPV in BALB/c mice.

Because IL-18 was not required for the infectivity of HMPV, we determined if the implication of NLRP3 inflammasome in the pathogenesis of this virus could be associated with the release of IL-1β. Therefore, C57BL/6 (IL-1β^+/+^) and IL-1β^-/-^ mice were infected with HMPV at a LD_50_ dose. MCC950-treated IL-1β^+/+^ and untreated IL-1β^-/-^ mice showed less weight loss and mortality, reduced IFN-γ and total protein levels, as well as leukocyte number in BAL, as compared to IL-1β^+/+^ mice without MCC950 treatment on day 5 post-infection (Fig 5B–5E). Other parameters including IL-1β, IL-6, TNF-α, IL-18, leukocyte differentiation and viral replication did not differ between IL-1β^+/+^ mice, IL-1β^-/-^ and IL-1β^+/+^ treated with MCC950 mice (S4 Fig). Thus, the involvement of NLRP3 inflammasome in the pathogenesis of HMPV seems to predominantly occur via IL-1β secretion.

### NLRP3 inflammasome activation is triggered by the HMPV SH protein

**Fig 6:**
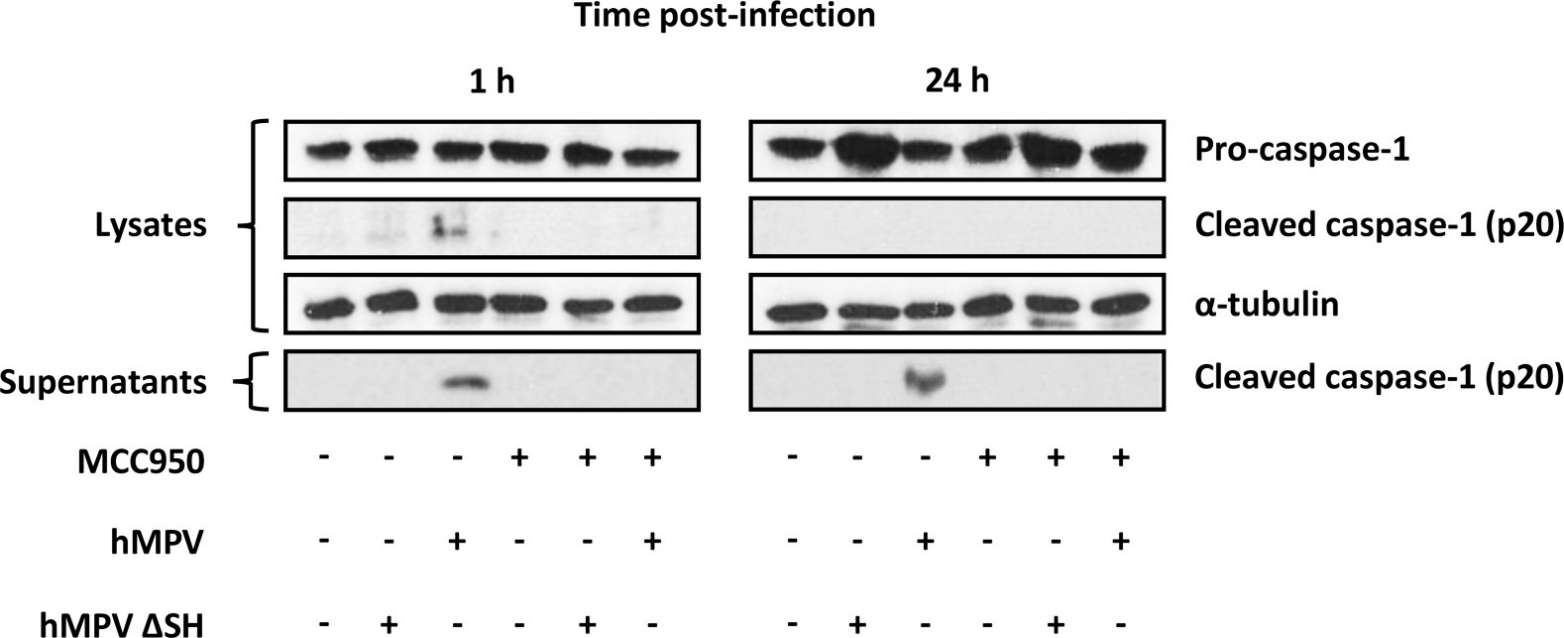
Cleavage of pro-caspase-1 into caspase-1 by HMPV. THP-1 cells were differenciated into macrophages using PMA (100 ng/ml) before treatment with 10 μM of MCC950 or DMSO (control) and then infected or not with HMPV or HMPV ΔSH at a MOI of 0.001. At 1 or 24 h post-infection, cell lysates and supernatants were harvested for Western Blot analysis of caspase-1 activation. The results were representative of three independent experiments.

To investigate whether HMPV SH protein is responsible for NLRP3 inflammasome activation, we first designed and generated HMPV ΔSH virus from strain C85473. We then evaluated caspase-1 cleavage as a marker of NLRP3 inflammasome activation because caspase-1 cleavage depends on the assembly of NLRP3, ASC and procaspase-1 to form inflammasome [5]. Western Blot analysis showed that HMPV inoculation induced caspase-1 cleavage in THP-1 cells (Fig 6). This cleavage, however, was prevented by MCC950 treatment. These results confirm the capacity of HMPV to induce NLRP3 inflammasome. In parallel, we found that HMPV ΔSH could not induce caspase-1 cleavage in THP-1 cells (Fig 6). Thus, SH protein seems to be the viral component triggering NLRP3 inflammasome activation, but other experiments are needed to determine if the mutant virus simply becomes inaccessible to pro-inflammatory danger sensors via compartmentalization or if it is physically unable to prime or activate the inflammasome. Cleaved caspase-1 was detected in both cell lysates and supernatants at 1 h but was only present in the cell supernatants at 24 h post-infection. This indicates that caspase-1 was rapidly cleaved and released into the supernatants [33] upon HMPV inoculation.

**Fig 7:**
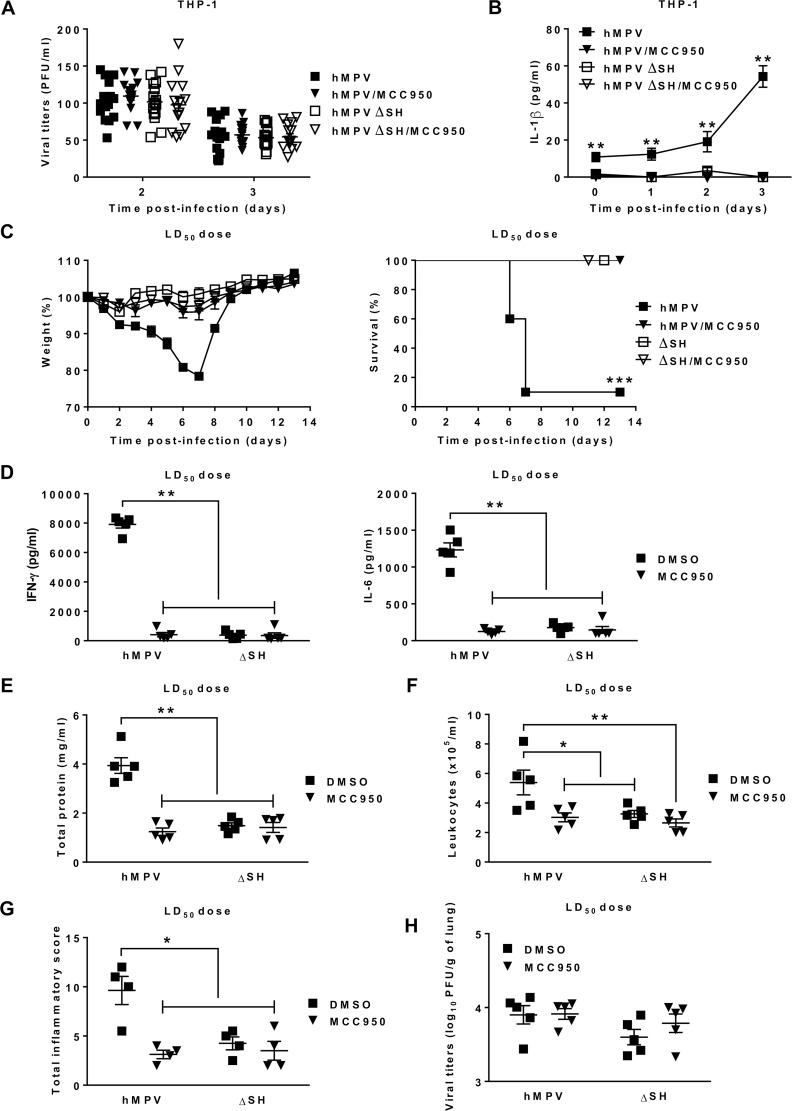
Lack of SH protein promotes attenuated pathogenicity of HMPV. THP-1 cells were differenciated into macrophages using PMA (100 ng/ml) before treatment with 10 μM of MCC950 or DMSO (control) and then infected or not with HMPV or HMPV ΔSH at a MOI of 0.001. (A) The viral titers were determined by immunostaining. Data were collected from three independent experiments. Values are shown as mean ± S.E.M (unpaired Student t-test, n = 5 per group per experiment). (B) IL-1β levels were measured in the cell supernatants. Values are shown as mean ± S.E.M (Kruskall-Wallis test followed by Dunn’s post hoc, ** *P* < 0.01 HMPV/MCC950, HMPVΔSH or HMPV ΔSH/MCC950 *vs* HMPV, n = 5 per group). (C) BALB/c mice were inoculated or not with HMPV at a LD_50_ dose (5 x 10^5^ PFU per mouse) or HMPV ΔSH in the presence or absence of MCC950 (5 mg/kg). MCC950 treatment was repeated for the next two days (1 time/day). The animals were monitored for survival and weight loss for 14 days after infection and euthanized if humane end points were reached (≤ 20% of initial weight). Values are shown as mean of weight ± S.E.M or average percent survival (Kaplan Meier survial curves, *** *P* < 0.001 HMPV/MCC950, HMPVΔSH or HMPV ΔSH/MCC950 *vs* HMPV, n = 10 per group). The lungs and BAL were harvested on day 5 post-infection. (D-F) IFN-γ, IL-6, total protein and leukocyte recruitment were evaluated in BAL (n = 5 per group). (G) histopathology was assessed in the lungs (n = 4 per group). Values are shown as mean ± S.E.M (Kruskall-Wallis test followed by Dunn’s post hoc, * *P* < 0.05; ** *P* < 0.01 HMPV/MCC950, HMPVΔSH or HMPV ΔSH/MCC950 *vs* HMPV). (H) Viral titers were evaluated in lung homogenates. Values are shown as mean ± S.E.M (Kruskall-Wallis test followed by Dunn’s post hoc, n = 5 per group).

We also investigated if the absence of SH protein could attenuate the infectivity of HMPV. No difference in viral replication between HMPV and HMPV ΔSH was observed in THP-1 cells (Fig 7A) but IL-1β production was abrogated in HMPV ΔSH-infected THP-1 cells (Fig 7B). The same tendency was observed during *in vivo* studies. Indeed, at the image of MCC950-treated mice, no mortality was seen in HMPV ΔSH-infected mice whereas slight weight loss as well as reduced IFN-γ, IL-6, total protein levels, leukocyte numbers in BAL and lung histopathological scores were observed, compared to HMPV-infected mice on day 5 post-infection (Fig 7C–7G & S5A Fig). No difference in IL-1β and TNF-α levels as well as leukocyte differentiation was observed on day 5 between HMPV ΔSH- and HMPV-infected mice (S5B and S5C Fig). However, a significant decrease of IL-1β was seen on day 1 in the HMPV ΔSH group (Fig 3). Thus, both NLRP3 inflammasome inhibition and SH protein deletion attenuated inflammation and lung injury. Moreover, SH protein did not impact on viral replication [34, 35], as demonstrated by no difference in the viral loads of lungs between and HMPV ΔSH- and HMPV-infected groups (Fig 7H). Altogether, we conclude that in the case of HMPV infection, NLRP3 inflammasome activation is triggered by the viral SH protein.

## Discussion

Our study clearly shows the role of the inflammasome and in particular IL-1β in the pathogenesis of HMPV using a pharmacological approach, BMDM NLRP3 KO cells and IL-1β^-/-^ mice. As a crucial component of the innate immune system, NLRP3 inflammasome serves an important role in host defense by recognizing RNA viral pathogens and triggering immune responses [36]. Although NLRP3 inflammasome has been reported to be implicated in many RNA viral diseases with distinct functions [7, 8], little is known about the involvement of this protein complex in the pathogenesis of HMPV. In such a context, this present study shows for the first time that NLRP3 inflammasome plays a detrimental role during HMPV infection and that such effect is mediated by the viral SH protein.

The contribution of NLRP3 inflammasome in the pathogenesis of RNA viruses occurs through its role as a trigger of only inflammation [12, 15, 18, 19] or both inflammation and viral replication [9, 10, 16, 17, 37]. Herein, we demonstrate that the involvement of NLRP3 inflammasome in the pathogenesis of HMPV only proceeds via its pro-inflammatory effect. Indeed, both *in vitro* and *in vivo* studies showed that viral replication was almost unaltered whereas inflammation was attenuated upon NLRP3 inflammasome inhibition during HMPV infection. This finding is consistent with two other studies which have also shown that NLRP3 inflammasome did not impact on viral replication during influenza and chikungunya diseases [21, 38]. Nevertheless, the suppression of NLRP3 inflammasome has been demonstrated to decrease fulminant hepatitis and Zika virus replication [10, 13] but increase Newcastle virus replication [9]. In other words, NLRP3 inflammasome plays distinct roles in the replication of RNA viruses.

NLRP3 inflammasome, once activated, will promote caspase-1-induced IL-1β and IL-18 maturation [29], but not other cytokines. In this study, NLRP3 inflammasome-independent secretion of IL-6 was observed in HMPV-infected J774.2 cells and TNF-α in THP-1 and J774.2 cells. This finding is consolidated by a recent *in vitro* study investigating RSV [39], the closest virus related to HMPV [40] also belonging to the *Pneumoviridae* family [22]. The authors reported that the secretion of IL-1β, not IL-6 was triggered by RSV-induced NLRP3 activation in primary human lung epithelial cells.

Although NLRP3 inflammasome is responsible for the secretion of only IL-1β and IL-18 in infected cells, the inhibition of this protein complex decreased the levels of not only IL-1β but also IL-6, TNF-α and IFN-γ in HMPV-infected mice. Furthermore, three other inflammatory parameters including the alteration of pulmonary capillary permeability, leukocyte recruitment and lung histopathological scores were also decreased upon NLRP3 inflammasome inhibition. These data are not unique and they are consistent with several previous reports [17–19, 21, 26, 38]. Thus, NLRP3 inflammasome may impact not only IL-1β and IL-18 secretion but also exert proinflammatory effects via unknown pathways [18] during RNA viral diseases in general and HMPV infection in particular.

To explain the proinflammatory function of NLRP3 inflammasome, it has been suggested that its activation may occur in concert with other proinflammatory pathways such as lipotoxicity-, oxidative stress- and TLR4-related pathways [41]. We suggest that NLRP3 inflammasome exerts pro-inflammatory effect during HMPV infection through biological activities of IL-1β. Indeed, this cytokine has been identified as an important regulator of inflammation, as evidenced by its capacity to stimulate neutrophil and macrophage recruitment and infiltration in some conditions [42, 43] and induce lung vascular permeability damage [44]. IL-1β has also been identified as an activator of IL-6 and IL-8 production [45, 46]. Most importantly, we reported that both NLRP3 inflammasome inhibition (BALB/c and C57BL/6 mice) and deletion of the gene encoding IL-1β (C57BL/6 mice) induced less weight loss with decreased mortality and inflammation in HMPV-infected mice. Moreover, the protective effect against HMPV disease did not differ between NLRP3 inflammasome inhibition and IL-1β deletion (C57BL/6 mice). Briefly, NLRP3 inflammasome-induced IL-1β release plays a crucial role during HMPV infection, at least in mice.

In the case of BALB/c mice, no mortality was found in animals infected with HMPV at a LD_50_ dose and treated with MCC950 (Fig 2A). By contrast, some mortality (25%) was detected in infected C57BL/6 mice given MCC950 treatment (Fig 5B). Furthermore, NLRP3 inflammasome inhibition was found to decrease IL-6 and IFN-γ levels in BAL from BALB/c mice (Fig 3) but only IFN-γ levels in the case of C57BL/6 mice on day 5 post-infection (Fig 5C). In parallel, IL-6, IFN-γ and TNF-α levels in BAL from BALB/c mice were strongly higher than those from C57BL/6 mice (Figs 3 and 5C & S4A Fig). All these findings indicate that HMPV-induced inflammation is more severe in BALB/c than C57BL/6 mice and that the role of NLRP3 inflammasome and IL-1β is more important in the former mice during HMPV infection. The different susceptibility of these two murine strains to HMPV [47] is a possible explanation.

Although NLRP3 inflammasome activation triggers the maturation of IL-1β and IL-18, we show that only IL-1β is involved in the pathogenesis of HMPV. Moreover, this involvement occurs at an early stage of infection process because IL-1β levels were only decreased upon NLRP3 inhibitor treatment on day 1 post-infection. This finding is not surprising since IL-1β as well as other IL-1 family cytokines are widely considered as early-response cytokines as they are released in the earliest stage of an immune response [48]. By contrast, IL-18 secretion was unaltered during infection and had no influence on the pathogenicity of HMPV in mice. This might be explained by the limited presence of macrophages during HMPV infections, which were shown to be an abundant source of IL-18 during *in vitro* studies [49] (Fig 1B). By contrast, polymorphonuclear neutrophils were abundant on day 1 and then lymphocytes were dominant on days 3 and 5 post-infection (Fig 4C).

NLRP3 inflammasome activation generally employs a two-step mechanism. In general, the first signal permitting the generation of pro-IL-1β and pro-IL-18 is triggered by the recognition of viral pathogens by Toll-like receptors (TLRs) or retinoic acid-inducible gene-I-like receptors [5]. Herein, we did not investigate the mechanisms by which the first signal occurs. However, we think that the TLR4 receptor may be responsible for this process because a recent study has demonstrated that among various TLRs including TLR2, TLR3, TLR4, TLR7 and TLR8, only TLR4 provides the first signal of NLRP3 inflammasome activation in RSV-infected lung epithelial cells [39]. Furthermore, TLR4^-/-^ mice induced less weight loss, decreased inflammation and no difference in viral replication, as compared to wild-type mice during HMPV infection [50]. These findings are consistent with our results when using either IL-1β^-/-^ mice or pharmacological approach for blocking NLRP3 inflammasome activation.

Recently, it has been shown that RNA viruses trigger NLRP3 inflammasome activation through a receptor interacting protein (RIP) 1/RIP3/dynamin-related protein 1 signaling pathway [51, 52]. Briefly, RNA virus infection initiates the assembly of RIP1/RIP3 complex, promoting activation of dynamin-related protein 1 and its translocation to mitochondria. This results in mitochondria damage, excessive reactive oxygen species generation and subsequent NLRP3 inflammasome activation [6]. RNA viral components responsible for triggering this pathway have been identified in several viral infections such as influenza virus M2 and PB1-F2 proteins [53–55], Measles virus V protein [56], RSV SH protein [39], encephalomyocarditis virus and rhinovirus 2B proteins [57, 58], coronavirus E protein [59] and enterovirus 71 3D protein [60]. Among these proteins, encephalomyocarditis virus and rhinovirus 2B proteins as well as RSV SH protein were recognized as viroporins. Viroporins from RNA viruses have been reported to be responsible for mitochondrial alteration [61]. Furthermore, once inserted on host cell membrane, viroporin will enable virus to tune ion permeability to stimulate a variety of viral cycle stages [62]. Taken together, we hypothesized that HMPV SH protein may be an activator of NLRP3 inflammasome during HMPV disease because it has been suggested to act as a viroporin [63] and the genomic structure of HMPV is closely related to that of RSV [40].

HMPV ΔSH viruses have been previously reported to be generated from the CAN97-83 (group A) or NL/1/99 (group B) strains [34, 35]. Here, we generated HMPV ΔSH using C85473 strain (group A) to verify our hypothesis [64]. In addition to the blockade of caspase-1 cleavage, a reliable marker of NLRP3 inflammasome activation, resulting from the lack of SH protein or MCC950 treatment, we found that identically to MCC950 treatment, SH deletion had no effect on the viral replication both *in vitro* and *in vivo*. This finding is consolidated by previous studies using other HMPV ΔSH viruses [34, 35]. We also detected that HMPV ΔSH-infected mice were protected against severe infection, as evidenced by no mortality, less weight loss and reduced inflammation. The evolution of HMPV infections was not different between HMPV ΔSH- and wild-type HMPV-infected mice receiving MCC950 treatment, but we acknowledge that no measures were taken to detect defective interfering particles in both viral preparations. In parallel, NLRP3 inflammasome inhibition had no influence on the pathogenesis of HMPV ΔSH. Taken together, we conclude that HMPV SH protein might be an activator of NLRP3 inflammasome in addition to its identified other roles to modulate type I IFN signaling pathway [65, 66], deteriorate cell host membrane permeability, regulate viral fusogenic function [63] and reduce CD4+ T cell activation [67].

In summary, we report for the first time a detrimental role of NLRP3 inflammasome during HMPV infection in murine models. Mechanistically, HMPV SH protein triggers NLRP3 inflammasome activation, leading to the cleavage of pro-IL-1β to form mature IL-1β. Although this cytokine is not crucial for controlling viral replication, it plays a major role in inflammatory process which is identified as an important feature for the pathogenicity of HMPV. Thus, the involvement of NLRP3 inflammasome in HMPV disease occurs via IL-1β-related inflammatory process rather than virus replication. In such a context, we believe that anti-inflammatory treatments in general and anti-IL-1β drugs in particular (i.e. the use of NLRP3 inhibitors) may be considered as novel potential strategies for the prevention and treatment of HMPV disease.

## Materials and methods

### Ethics statement

Six-week old female BALB/c mice with a body weight of 16.5–18 g were purchased from Charles River Laboratories (Senneville, QC, Canada). IL-1β^-/-^ mice were kindly provided by Dr Steve Lacroix (Infectious Disease Research Centre, Quebec City, QC, Canada). Age-matched wild-type C57BL/6 mice were purchased from Charles River Laboratories. Mice were housed under pathogen-free conditions in the animal research facility of the Quebec University Health Centre (Quebec City, QC, Canada) and allowed to acclimatize for one week prior to the start of experiments.

All experimental procedures with mice were approved by the Animal Protection Committee of the Quebec University Health Centre in accordance with guidelines of the Canadian Council on Animal Care (Protocol number: CPAC 2017-139-1). Before inoculation of substances or euthanasia, mice were anaesthetized by inhalation of isoflurane vaporized at concentrations of 3–4% and oxygen flow rate adjusted to 1.5 l/min. Individual body weight and clinical signs were used to monitor animal health and response to infection and were recorded daily. Mice were euthanized by CO_2_ inhalation upon loss of 20% of initial body weight.

### Cells and viruses

LLC-MK2 cells (ATCC, Manassas, VA, USA) were maintained in minimal essential medium (Thermo Fisher Scientific, Burlington, ON, Canada) supplemented with 10% fetal bovine serum (FBS) (Wisent, Saint-Jean-Baptiste, QC, Canada) and HEPES buffer (2.5 g/l). Murine macrophage J774.2 cells were kindly provided by Dr Sachiko Sato (Infectious Disease Research Centre, Quebec City, QC, Canada) and maintained in Dulbecco’s modified eagle medium (Thermo Fisher Scientific) supplemented with 10% FBS and 1% penicillin-streptomycin (Thermo Fisher Scientific). Human monocyte-like THP-1 cells were generously provided by Dr Francesca Cicchetti (Quebec University Health Centre, Quebec City, QC, Canada) and maintained in RPMI 1640 medium (Thermo Fisher Scientific) supplemented with 10% FBS, 1% penicillin-streptomycin, 1% non-essential amino acids (Thermo Fisher Scientific) and 0.05 mM 2-mercaptoethanol (Sigma Aldrich, Oakville, ON, Canada). Differentiation of THP-1 cells into macrophages by the addition of phorbol 12-myristate 13-acetate (100 ng/ml) [68] (Sigma Aldrich) was carried out in all *in vitro* experiments. Immortalized murine bone-marrow derived macrophages WT (BMDM iWT) or NLRP3 -/- (BMDM iNLRP3KO) were kindly provided by Dr Bénédicte Py (Centre International de Recherche en Infectiologie CIRI, Lyon, France) and maintained in Dulbecco’s modified eagle medium (Thermo Fisher Scientific) high glucose, supplemented with 10% FBS and 1% penicillin-streptomycin (Thermo Fisher Scientific).

The HMPV strain C85473, a clinical isolate, and the recombinant HMPV strain C85473 ΔSH were grown in LLC-MK2 cells and concentrated as previously described [69]. Viruses were concentrated by ultracentrifugation and pellets resuspended in PBS. Viral stocks were sequenced and titers were determined by immunostaining [70] and expressed as plaque-forming units (PFU) per milliliter.

### Construction of SH gene deletion mutant cDNA and HMPV ΔSH recovery

The strategy for the construction of the plasmid encoding the full-length genomic cDNA of HMPV A1/C-85473 strain (GenBank accession number KM408076.1) and the subsequent production of recombinant viruses is described in details in [64]. Briefly, the full-length genomic cDNA of HMPV A1/C-85473 strain was generated by RT-PCR using the Superscript II reverse transcriptase (Thermo Fisher Scientific) and amplified by Phusion DNA polymerase (New England Biolabs, Whitby, ON, Canada). A Gibson Assembly (Cloning Kit, New England Biolabs) was performed to integrate the genomic viral cDNA into a pSP72 plasmid (Promega, Madison, WI, USA) containing a T7 terminator, the hepatitis delta virus (HDV) ribozyme and a T7 promoter. To generate HMPV ΔSH virus, the mentioned plasmid was amplified using specific primers, designed to match before the SH gene start sequence (5’-GGGACAAGTAGTTATGGA-3’) and after the intergenic SH-G region (5’-ACTCTGATGTGTTTTTACTAAC-3’), in order to extract completely the SH gene sequence. After amplification, linear DNA was phosphorylated and ligated with the T4 Ligase to re-circularize the shortened HMPV genome. The newly generated HMPV ΔSH genomic plasmid was validated by complete sequencing prior to transfection. BSR-T7 cells were then co-transfected (Lipofectamin 2000, Thermo Fisher Scientific) with the HMPV ΔSH genomic plasmid and 4 supporting plasmids expressing the N, P, L, and M2.1 viral ORFs. Seven hours after transfection, the medium was replaced by Opti-MEM supplemented with 1% Non-Essential Amino Acids (Thermo Fisher Scientific). Transfected cells were incubated at 37°C and 5% CO2 for four days. At this point, LLC-MK2 cells were added to the transfected BSR T7 cells and co-cultured at 37°C and 5% CO2 with the addition of fresh trypsin (0.0002%) after two days. Two or three days after co-culture, cells were harvested, sonicated and centrifuged at 2000 x g for 5 min at room temperature. The supernatant was collected, diluted into infection medium (Opti-MEM supplemented with 0.0002% trypsin), and inoculated onto confluent LLC-MK2 monolayers. Infected monolayers were monitored for the appearance of characteristic cytopathic effect, and the harvested virus was further amplified through serial passages in LLC-MK2 cells.

### Assessment of cytotoxicity

The CC_50_ concentration of NLRP3 inhibitor MCC950 (Tocris Bioscience, Bristol, UK) was determined in J774.2 and THP-1 cells using the CellTiter 96 Aqueous One Solution Cell Proliferation Assay (Promega) according to the manufacturer’s instructions.

### *In vitro* viral infections

J774.2 or THP-1 cells were treated with 10 μM of MCC950 [26] and incubated at 37°C for 1.5 h. Equivalent dilutions of dimethyl sulfoxide (DMSO) (Sigma Aldrich) served as control. The cells were then inoculated with wild-type HMPV or HMPV ΔSH at a MOI of 0.001 (THP-1) or 0.01 (J774.2) per well and incubated at 37°C. Cell lysates and supernatants were harvested at 1; 24; 48 and 72 h post-infection for ELISA or Western Blot analyses. In addition, the viral titers were determined by immunostaining three days post-infection and expressed as PFU per milliliter. BMDM iWT and iNLRP3KO cells in 24-well plates were washed in PBS and inoculated with Opti-MEM (mock), wild-type HMPV or ΔSH HMPV at a MOI of 0.1 in Opti-MEM + 0.0002% trypsin. After 3 h adsorption at 37°C, inocula were removed and changed by fresh cell culture medium DMEM high glucose + 10% FBS and 1% penicillin-streptomycin (Thermo Fisher Scientific). Cell supernatants were harvested at 1, 24, 48 and 72 h post-infection to perform IL-1β or TNF-α quantification by ELISA assays (DuoSet ELISA, R&D Systems), according to manufacturer’s instructions, and viral titrations, as previously described [70].

### *In vivo* viral infections, NLRP3 inflammasome inhibition and IL-18 inhibition

A preliminary study has been carried out to determine the sublethal, LD_50_ and lethal doses of virus in mice. BALB/c mice were inoculated intranasally with HMPV strain C85473 (sublethal dose = 3 x 10^5^; LD_50_ = 5 x 10^5^ or lethal dose = 10^6^ PFU per mouse) or HMPV ΔSH whereas IL-1β^-/-^ and wild-type C57BL/6 mice were inoculated with 2 x 10^6^ PFU of HMPV strain C85473. The LD_50_ dose in C57BL/6 mice was four-fold higher than that of BALB/c mice because C57BL/6 mice are less susceptible to HMPV infection, as compared to BALB/c mice [47]. Equal volumes of Opti-MEM medium served as mock infection.

To block NLRP3 inflammasome activation, MCC950 (5 mg/kg) [21, 71] was mixed and administered intranasally at the same time with the virus. However, MCC950 was also given 24 h post-infection in a single experiment. Equivalent dilutions of DMSO (Sigma Aldrich) served as control. This treatment was repeated once a day for two consecutive days. For IL-18 inhibition, immediately following inoculation of virus, mice underwent intraperitoneal injections of IL-18BP at a dose of 75 μg/kg (R&D Systems, Minneapolis, MN, USA) [72]. This treatment was repeated once a day for two consecutive days during infections. Control mice were given sterile saline in a similar manner.

### Lung viral titrations

To evaluate viral titers on days 1, 3 and 5 post-infection, mice were euthanized and whole lungs were harvested and then homogenized in PBS (1 ml/sample) using TH Tissue Homogenizer (Omni International, Kennesaw, GA, USA). Supernatants were collected after centrifugation at 350 x *g* for 10 minutes at 4°C and methylcellulose was used to determine viral titers by immunostaining and expressed as PFU per gram of lung.

### Broncho-alveolar lavage and cell counting

On days 1, 3 and 5 post-infection, mice were euthanized and broncho-alveolar lavage (BAL) was performed with sterile cold phosphate-buffered saline (PBS). The cells in the lavage fluid were pelleted by centrifugation at 300 x *g* for 5 min at 4°C, and then suspended in PBS whereas BAL supernatants were collected for evaluating other inflammatory parameters. Viable cell number was determined using a hemocytometer and expressed as number per milliliter of BAL.

For differential cell counts, 100 μl of suspended cells were spun onto a slide by using a Shandon Cytospin 3 cytocentrifuge (Thermo Fisher Scientific) at 100 x *g* for 5 min at room temperature. Slides were then air-dried and stained with May-Grunewald Giemsa solutions (Sigma Aldrich) according to the manufacturer’s instructions. Differential cell counts were made with standard morphological criteria by counting at least 300 cells per sample. The results were expressed as differential percentage.

### BAL cytokine and total protein quantification

The concentrations of IL-1β, IL-6, TNF-α, IFN-γ and IL-18 in the cell supernatants or BAL fluids were determined using the Mouse or Human IL-1β, IL-6, TNF-α, IFN-γ, IL-18 DuoSet ELISA (R&D Systems) or the Mouse IL18/IL-18 ELISA Pair Set (Sino Biological, Beijing, China) according to the manufacturer’s instructions. The results were expressed as picogram per milliliter of BAL.

Total protein levels in the BAL supernatants were determined using Quick Start Bradford Protein Assay (Bio-Rad Laboratories, Mississauga, ON, Canada) according to the manufacturer’s instructions. The results were expressed as milligram per milliliter of BAL.

### FACS studies of lung immune cells

In order to analyze lung-infiltrating immune cells, mice were deeply anesthetized and perfused intracardially with D-PBS without Ca2+ and Mg2+ prior to (day 0) and on days 1, 3 and 5 post-infection. Whole lungs were collected and digested with Liberase TL (Roche Diagnostics, Mannheim, Germany). Lung homogenates were incubated for 1 h at 37°C then filtered through a 70-**μ**m cell strainer (BD Biosciences, Mississaugo, ON, Canada). The cell suspension was centrifuged at 300 x g for 10 min at room temperature. The supernatant was aspirated and cells were washed twice with D-PBS plus 2% FBS. Cells were first incubated on ice for 30 min with fixable viability stain 510 (BD Biosciences, CA, USA), then washed and incubated again on ice for 30 min with purified rat anti–mouse CD16/CD32 (Mouse Fc Block; BD Biosciences, CA, USA). Red blood cells were lysed with BD Pharm Lyse (RBC Lysis Buffer 10X –BioLegend, San Diego, CA, USA) and the recovered leukocytes were washed and resuspended in D-PBS. After the washing step, cells were incubated on ice for 40 min with a pool of antibodies (anti-CD45, anti-CD11b, anti-CD170 (Siglec-F), anti-Ly6C, anti-Ly6G, anti-CD11c, anti-CD115, anti-B220, anti-CD3ε, anti-CD4 and anti-CD8a /BD Bioscience, CA, USA). Number of cells was determined with Precision Count Beads (BioLegend, San Diego, CA, USA). Labeled cells were then washed and resuspended in DPBS. Flow cytometry analyses and data acquisition were performed by using a BD SORP LSR II and the BD FACSDiva software, respectively.

### Western blot analysis

The total proteins in cell supernatants were concentrated using Amicon Ultra-15 Centrifugal Filters (Millipore Canada, Etobicoke, ON, Canada) according to the manufacturer’s instructions. The concentrations of protein in cell lysates and supernatants were determined using Quick Start Bradford Protein Assay. Equal protein amounts were separated on 10% SDS-PAGE gels and then transferred to nitrocellulose membranes (GE HealthCare Life Sciences, Mississauga, ON, Canada) and blocked using 5% BSA (Sigma Aldrich). Primary antibodies were used at a dilution of 1:1,000 goat anti-caspase-1 (R&D Systems); rabbit anti-cleaved caspase-1 (p20) or mouse anti-α-tubulin (Cell Signaling Technology, Boston, MA, USA). Secondary antibodies were used at a dilution of 1:1,000 HRP-conjugated rabbit anti-goat (R&D systems) or 1:5,000 HRP-conjugated rabbit anti-mouse or mouse anti-rabbit (Cell Signaling Technology). Signal detection was carried out using the West Pico Plus Chemiluminescent Substrate (Thermo Fisher Scientific).

### Histological analysis

On day 5 post-infection, mice were euthanized and their lungs were removed. Tissue was fixed in 4% paraformaldehyde, embedded in paraffin, sectioned in slices of 5 **μ**m, and stained with hematoxylin and eosin. Slides were digitalized at 40X magnification using a Nanozoomer slide scanner (Hamamatsu, Japan) and scored using NDP viewer 2.0 software (Hamamatsu, Japan). The histopathological scores were determined by a pathologist and a medical biologist who were blinded to the experimental data. A semi-quantitative scale was used to score bronchial/endobronchial, peribronchial, perivascular, interstitial, pleural and intra-alveolar inflammation [73]. Scores represent consensus between the two observers. The results were expressed as lung total inflammatory scores.

### Statistical analysis

All statistical tests were conducted using the GraphPad Prism version 6.0 (GraphPad Software, La Jolla, CA, USA). The results were expressed as the mean ± S.E.M for each group and 'n' referred to the sample size. Survival data were analyzed by comparing Kaplan-Meier curves using the log-rank test. Viral titers, cytokines and total protein levels, immune cell recruitment, cell differentiation as well as lung histopathological scores were analyzed using unpaired Student t-test, Mann-Whitney U-test, one-way analysis of variance (ANOVA) followed by Tukey post hoc or Kruskal-Wallis test followed by Dunn’s post hoc for multiple comparisons. Differences were considered statistically significant when *P* < 0.05.

## Supporting information

S1 Fig Cytotoxicity of MCC950 in THP-1 and J774.2 cells. Cytotoxicity of MCC950 in THP-1 and J774.2 cells were assessed by the MTS test. The experiment was performed in triplicates.
(TIF)

S2 Fig HMPV- and HMPV ΔSH-infected BMDM and NLRP3 KO BMDM cells. Immortalized murine Bone-Marrow Derived-Macrophages WT (iWT) or NLRP3 *-/-* (iNLRP3KO) were infected with WT HMPV or ΔSH HMPV at a MOI of 0.1. (A) The viral titers were determined in PFU/ml from cell supernatants harvested after 1, 24, 48 or 72 hpi. Data were collected from duplicates. Values are shown as mean ± S.E.M. (B) IL-1β and TNF-α cytokines levels were measured in the cell supernatants by ELISA. Data were collected from duplicates. Values are shown as mean ± S.E.M.
(TIF)

S3 Fig Lymphocyte populations in lungs of HMPV- and HMPV-MCC950 treated-mice. Balb/c mice (n = 5 mice/group) were infected with HMPV at a LD_50_ dose. Immune cell infiltration in lung homogenates was evaluated by flow cytometry for different time points. (A) The percentage of total lymphocytes with respect to total pulmonary leukocytes on day 3 post-infection. (B) Three infiltrating subpopulations of total lymphocytes are represented. CD45 was used to discriminate lung-infiltrating leukocytes from whole living cells. B and T cells were selected according to B220 and CD3E expression, respectively. The expression levels of CD4 and CD8 were used to select T helper cells and cytotoxic T cells, among CD3E+ infiltrating T cells.
(TIF)

S4 Fig Cytokines and immune cell infiltration in C57BL/6 and IL-1β^-/-^ mice. IL-1β^-/-^ and C57BL/6 (IL-1β^+/+^) mice were inoculated with HMPV at a LD_50_ dose (2 x 10^6^ PFU per mouse) in the presence or absence of MCC950 (5 mg/kg). MCC950 treatment was repeated for the next two days (1 time/day). (A-B) Cytokines and leukocytes differentiation were evaluated in BAL on day 5 post-infection. (C) Viral titers were assessed in lung homogenates. Values are shown as mean ± S.E.M (ANOVA followed by Tukey post hoc, n = 10 per group).
(TIF)

S5 Fig Triggering of NLRP3 inflammasome activation by HMPV SH protein. BALB/c mice were inoculated or not with HMPV at a LD_50_ dose (5 x 10^5^ PFU per mouse) or HMPV ΔSH in the presence or absence of MCC950 (5 mg/kg). MCC950 treatment was repeated for the next two days (1 time/day). The lungs and BAL were harvested on day 5 post-infection. (A) Histopathology was assessed in the lungs and inflammatory scores are indicated in Fig 7G. (B) IL-1β and TNF-α levels were measured in BAL. (C) Cell differentiation was determined in BAL.
(TIF)
