# Supplementary figures and images for: Human metapneumovirus activates NOD-like receptor protein 3 inflammasome via its small hydrophobic protein which plays a detrimental role during infection in mice
Source: PLoS Pathog. 2019 Apr 9;15(4):e1007689. doi: 10.1371/journal.ppat.1007689 (PMC6474638; doi:10.1371/journal.ppat.1007689)

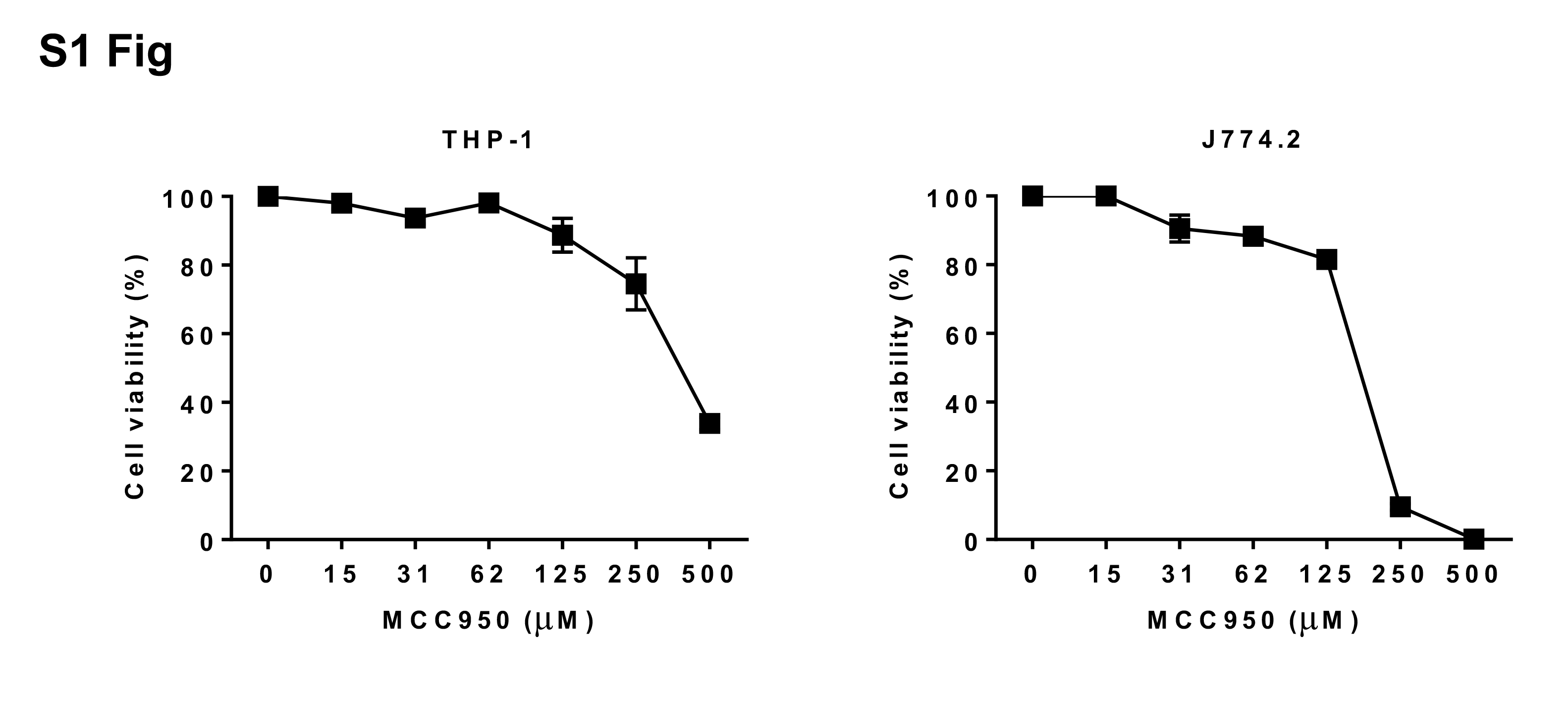

Supplement: S1 Fig — Cytotoxicity of MCC950 in THP-1 and J774.2 cells were assessed by the MTS test. The experiment was performed in triplicates. (TIF) [file ppat.1007689.s001.tif]

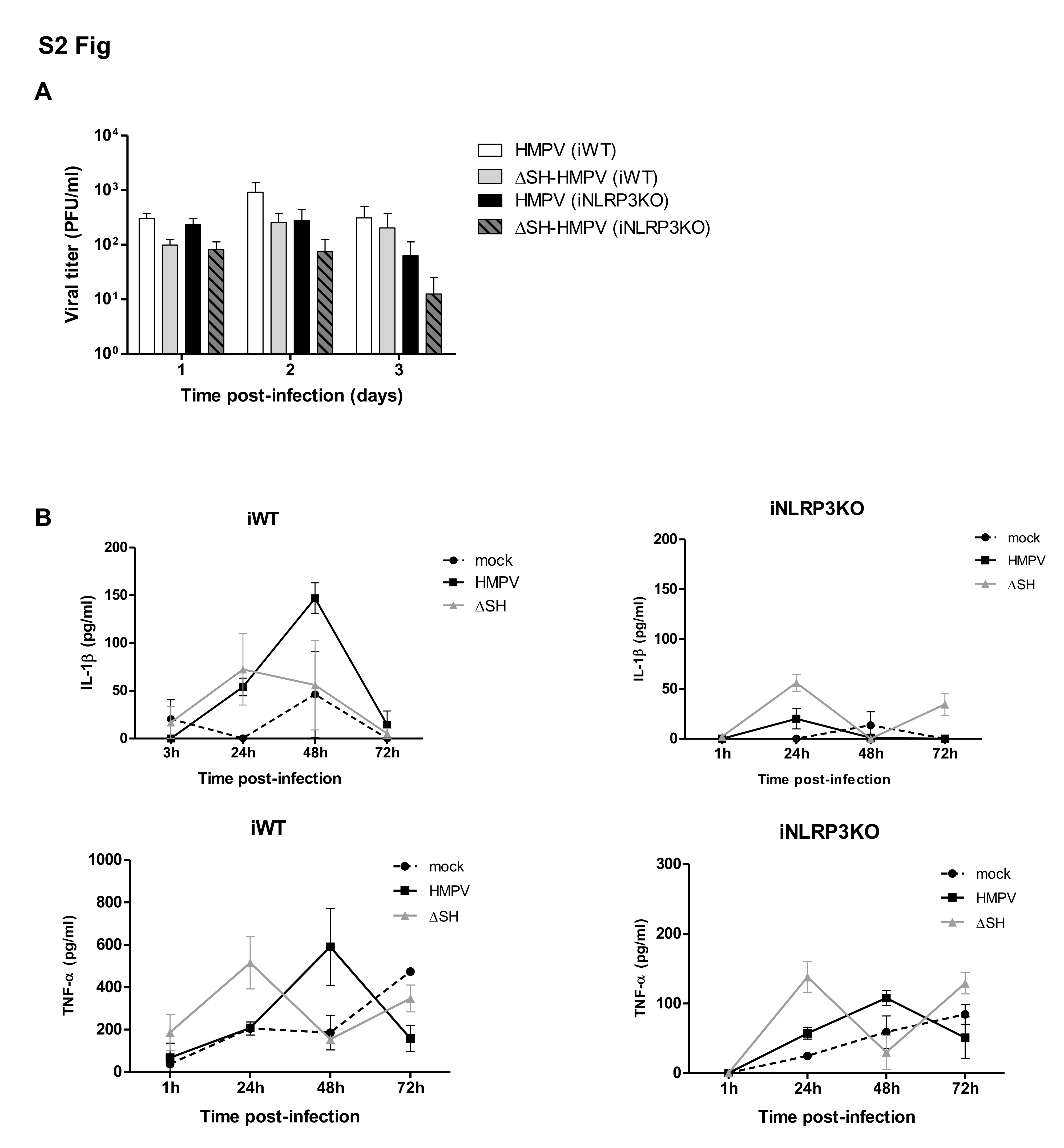

Supplement: S2 Fig — Immortalized murine Bone-Marrow Derived-Macrophages WT (iWT) or NLRP3 -/- (iNLRP3KO) were infected with WT HMPV or ΔSH HMPV at a MOI of 0.1. (A) The viral titers were determined in PFU/ml from cell supernatants harvested after 1, 24, 48 or 72 hpi. Data were collected from duplicates. Values are shown as mean ± S.E.M. (B) IL-1β and TNF-α cytokines levels were measured in the cell supernatants by ELISA. Data were collected from duplicates. Values are shown as mean ± S.E.M. (TIF) [file ppat.1007689.s002.tif]

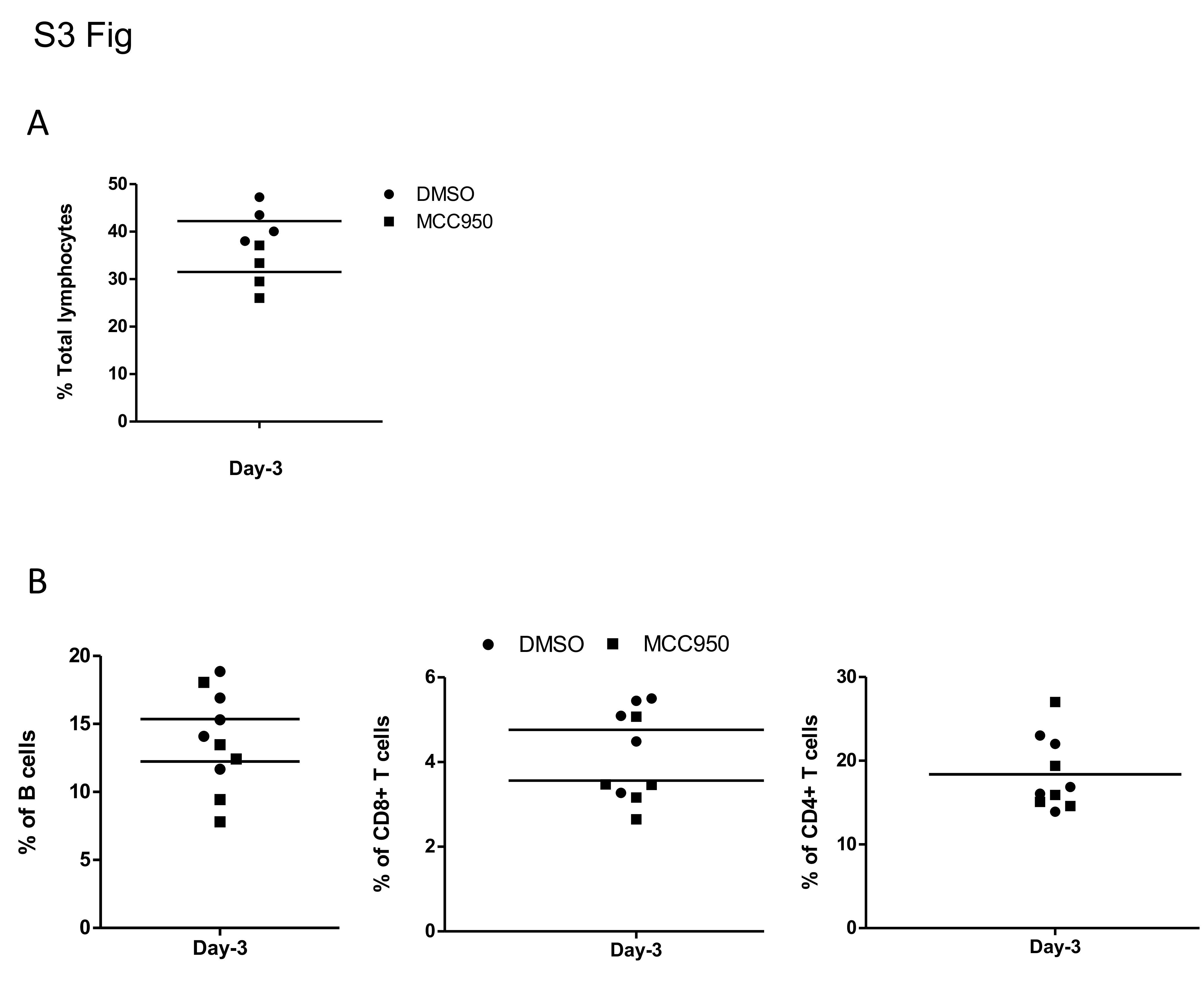

Supplement: S3 Fig — Balb/c mice (n = 5 mice/group) were infected with HMPV at a LD50 dose. Immune cell infiltration in lung homogenates was evaluated by flow cytometry for different time points. (A) The percentage of total lymphocytes with respect to total pulmonary leukocytes on day 3 post-infection. (B) Three infiltrating subpopulations of total lymphocytes are represented. CD45 was used to discriminate lung-infiltrating leukocytes from whole living cells. B and T cells were selected according to B220 and CD3E expression, respectively. The expression levels of CD4 and CD8 were used to select T helper cells and cytotoxic T cells, among CD3E+ infiltrating T cells. (TIF) [file ppat.1007689.s003.tif]

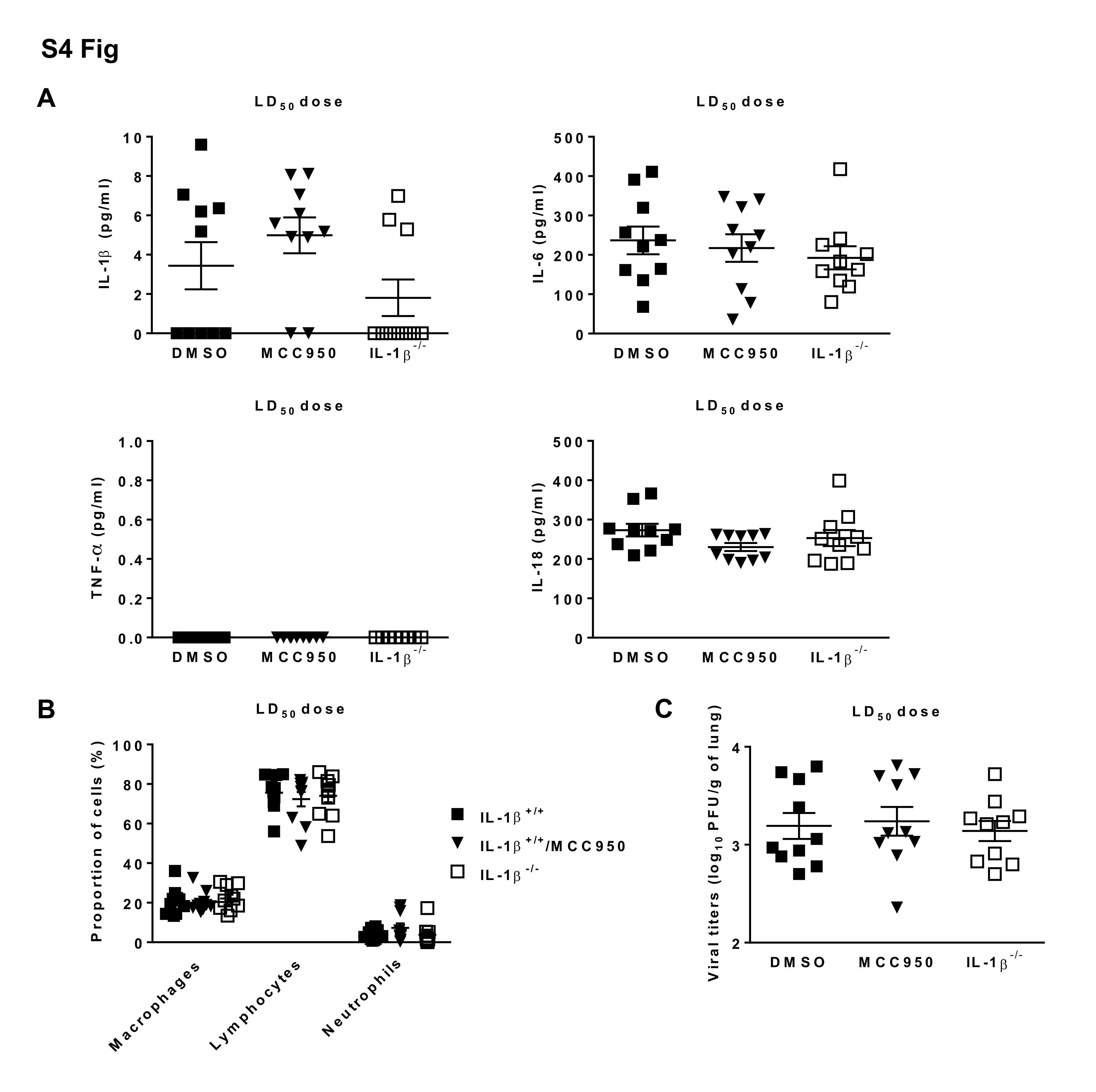

Supplement: S4 Fig — IL-1β-/- and C57BL/6 (IL-1β+/+) mice were inoculated with HMPV at a LD50 dose (2 x 10^6^ PFU per mouse) in the presence or absence of MCC950 (5 mg/kg). MCC950 treatment was repeated for the next two days (1 time/day). (A-B) Cytokines and leukocytes differentiation were evaluated in BAL on day 5 post-infection. (C) Viral titers were assessed in lung homogenates. Values are shown as mean ± S.E.M (ANOVA followed by Tukey post hoc, n = 10 per group). (TIF) [file ppat.1007689.s004.tif]

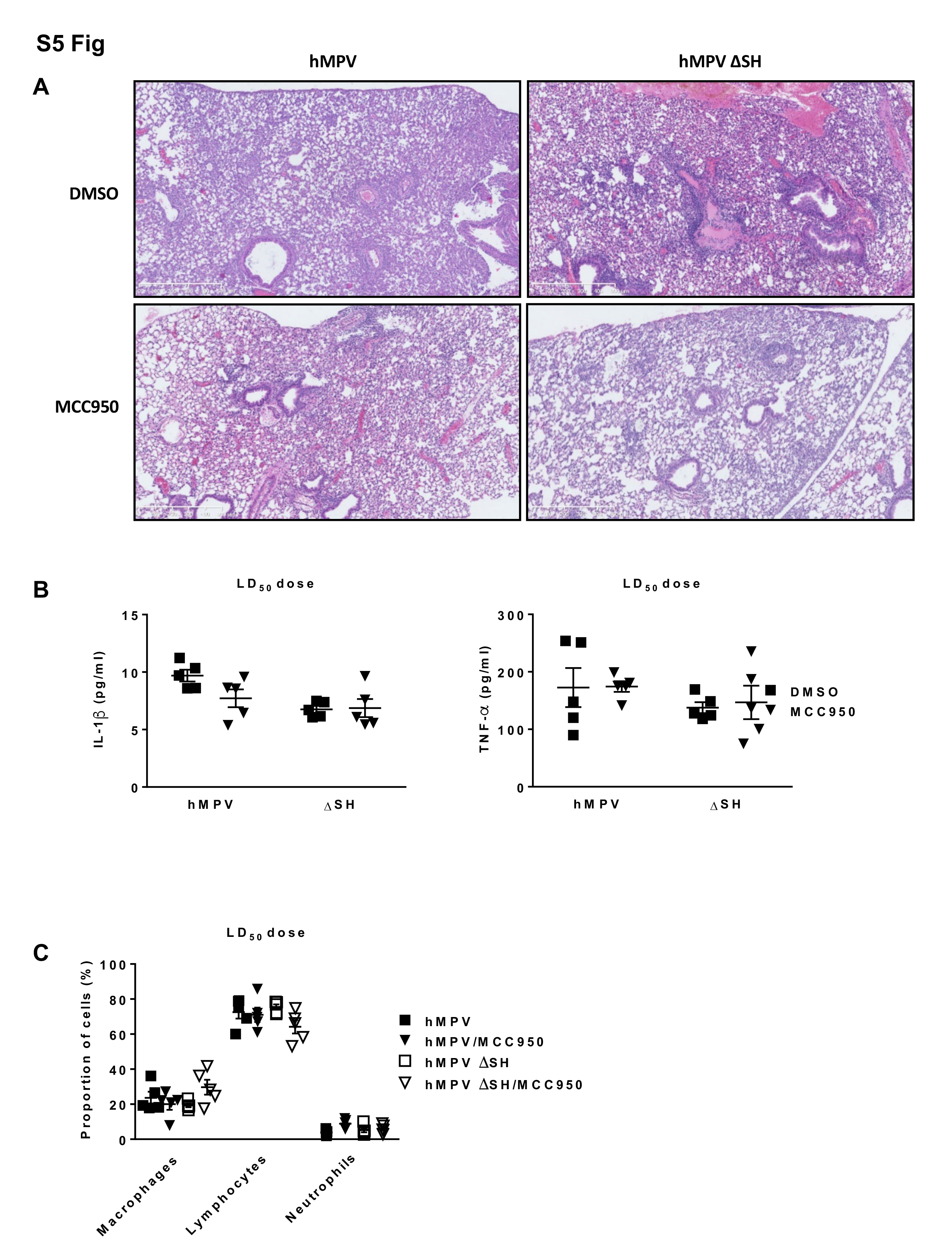

Supplement: S5 Fig — BALB/c mice were inoculated or not with HMPV at a LD50 dose (5 x 10^5^ PFU per mouse) or HMPV ΔSH in the presence or absence of MCC950 (5 mg/kg). MCC950 treatment was repeated for the next two days (1 time/day). The lungs and BAL were harvested on day 5 post-infection. (A) Histopathology was assessed in the lungs and inflammatory scores are indicated in Fig 7G. (B) IL-1β and TNF-α levels were measured in BAL. (C) Cell differentiation was determined in BAL. (TIF) [file ppat.1007689.s005.tif]
